# On complete convergence and complete moment convergence for weighted sums of $\rho^{*}$-mixing random variables

**DOI:** 10.1186/s13660-018-1710-2

**Published:** 2018-06-01

**Authors:** Pingyan Chen, Soo Hak Sung

**Affiliations:** 1 Department of Mathematics, Jinan University, Guangzhou, China; 2 Department of Applied Mathematics, Pai Chai University, Daejeon, South Korea

**Keywords:** 60F15, $\rho^{*}$-mixing random variables, Complete convergence, Complete moment convergence, Weighted sum

## Abstract

Let $r\geq1$, $1\leq p<2$, and $\alpha, \beta>0$ with $1/\alpha+1/\beta=1/p$. Let $\{a_{nk}, 1\leq k\leq n,n\geq1\}$ be an array of constants satisfying $\sup_{n\geq1}n^{-1}\sum^{n}_{k=1}|a_{nk}|^{\alpha}<\infty$, and let $\{ X_{n},n\geq1\}$ be a sequence of identically distributed $\rho^{*}$-mixing random variables. For each of the three cases $\alpha< rp$, $\alpha=rp$, and $\alpha>rp$, we provide moment conditions under which

$$\sum^{\infty}_{n=1}n^{r-2}P \Biggl\{ \max_{1\leq m\leq n} \Biggl\vert \sum^{m}_{k=1}a_{nk}X_{k} \Biggr\vert >\varepsilon n^{1/p} \Biggr\} < \infty,\quad \forall \varepsilon>0. $$

We also provide moment conditions under which

$$\sum^{\infty}_{n=1}n^{r-2-q/p} E \Biggl( \max_{1\leq m\leq n} \Biggl\vert \sum^{m}_{k=1}a_{nk}X_{k} \Biggr\vert -\varepsilon n^{1/p} \Biggr)_{+}^{q}< \infty,\quad \forall\varepsilon>0, $$

where $q>0$. Our results improve and generalize those of Sung (Discrete Dyn. Nat. Soc. 2010:630608, 2010) and Wu et al. (Stat. Probab. Lett. 127:55–66, 2017).

## Introduction

Due to the estimation of least squares regression coefficients in linear regression and nonparametric curve estimation, it is very interesting and meaningful to study the limit behaviors for the weighted sums of random variables.

We recall the concept of $\rho^{*}$-mixing random variables.

### Definition 1.1

Let $\{X_{n},n\geq1\}$ be a sequence of random variables defined on a probability space $(\Omega, {\mathcal {F}}, P)$. For any $S\subset N=\{1,2,\ldots\}$, define ${\mathcal {F}}_{S}=\sigma(X_{i}, i\in S)$. Given two *σ*-algebras ${\mathcal {A}}$ and ${\mathcal {B}}$ in ${\mathcal {F}}$, put

$$\rho({\mathcal {A}},{\mathcal {B}})=\sup \biggl\{ \frac{EXY-EXEY}{\sqrt {E(X-EX)^{2}E(Y-EY)^{2}}}: X\in L_{2}({\mathcal {A}}), Y\in L_{2}({\mathcal {B}}) \biggr\} . $$

Define the $\rho^{*}$-mixing coefficients by

$$\rho^{*}_{n}=\sup\bigl\{ \rho({\mathcal {F}}_{S}, {\mathcal {F}}_{T}): S, T\subset N \operatorname{ with }\operatorname{dist}(S,T) \geq n\bigr\} , $$

where $\operatorname{dist}(S,T)=\inf\{|s-t|: s\in S, t\in T\}$. Obviously, $0\leq\rho ^{*}_{n+1}\leq\rho^{*}_{n}\leq\rho^{*}_{0}=1$. Then the sequence $\{X_{n},n\geq1\}$ is called $\rho^{*}$-mixing if there exists $k\in N$ such that $\rho^{*}_{k}<1$.

A number of limit results for $\rho^{*}$-mixing sequences of random variables have been established by many authors. We refer to Bradley [3] for the central limit theorem, Bryc and Smolenski [4], Peligrad and Gut [5], and Utev and Peligrad [6] for the moment inequalities, and Sung [1] for the complete convergence of weighted sums.

Special cases for weighted sums have been studied by Bai and Cheng [7], Chen et al. [8], Choi and Sung [9], Chow [10], Cuzick [11], Sung [12], Thrum [13], and others. In this paper, we focus on the array weights $\{a_{nk}, 1\le k\le n, n\ge1\}$ of real numbers satisfying

1.1 $$ \sup_{n\geq1}n^{-1}\sum^{n}_{k=1}|a_{nk}|^{\alpha}< \infty $$

for some $\alpha>0$. In fact, under condition (1.1), many authors have studied the limit behaviors for the weighted sums of random variables.

Let $\{X, X_{n}, n\geq1\}$ be a sequence of independent and identically distributed random variables. When $\alpha=2$, Chow [10] showed that the Kolmogorov strong law of large numbers

1.2 $$ n^{-1}\sum^{n}_{k=1}a_{nk}X_{k} \rightarrow0 \quad \mbox{a.s.} $$

holds if $EX=0$ and $EX^{2}<\infty$. Cuzick [11] generalized Chow’s result by showing that (1.2) also holds if $EX=0$ and $E|X|^{\beta}<\infty$ for $\beta>0$ with $1/\alpha+1/\beta=1$. Bai and Cheng [7] proved that the Marcinkiewicz–Zygmund strong law of large numbers

1.3 $$ n^{-1/p}\sum^{n}_{k=1}a_{nk}X_{k} \rightarrow0 \quad \mbox{a.s.} $$

holds if $EX=0$ and $E|X|^{\beta}<\infty$, where $1\leq p<2$ and $1/\alpha +1/\beta=1/p$. Chen and Gan [14] showed that if $0< p<1$ and $E|X|^{\beta}<\infty$, then (1.3) still holds without the independent assumption.

Under condition (1.1), a convergence rate in the strong law of large numbers is also discussed. Chen [15] showed that

1.4 $$ \sum^{\infty}_{n=1}n^{r-2}P\Biggl\{ \max_{1\leq m\leq n} \Biggl\vert \sum^{m}_{k=1}a_{nk}X_{k} \Biggr\vert >\varepsilon n^{1/p}\Biggr\} < \infty,\quad \forall \varepsilon>0, $$

if $\{X, X_{n}, n\geq1\}$ is a sequence of identically distributed negatively associated (NA) random variables with $EX=0$ and $E|X|^{(r-1)\beta}<\infty$, where $r>1$, $1\leq p<2$, $1/\alpha+1/\beta=1/p$, and $\alpha< rp$. The main tool used in Chen [15] is the exponential inequality for NA random variables (see Theorem 3 in Shao [16]). Sung [1] proved (1.4) for a sequence of identically distributed $\rho^{*}$-mixing random variables with $EX=0$ and $E|X|^{rp}<\infty$, where $\alpha>rp$, by using the Rosenthal moment inequality. Since the Rosenthal moment inequality for NA has been established by Shao [16], it is easy to see that Sung’s result also holds for NA random variables. However, for $\rho^{*}$-mixing random variables, we do not know whether the corresponding exponential inequality holds or not, and so the method of Chen [15] does not work for $\rho^{*}$-mixing random variables. On the other hand, the method of Sung [1] is complex and not applicable to the case $\alpha\leq rp$.

In this paper, we show that (1.4) holds for a sequence of identically distributed $\rho^{*}$-mixing random variables with suitable moment conditions. The moment conditions for the cases $\alpha< rp$ and $\alpha>rp$ are optimal. The moment conditions for $\alpha=rp$ are nearly optimal. Although the main tool is the Rosenthal moment inequality for $\rho ^{*}$-mixing random variables, our method is simpler than that of Sung [1] even in the case $\alpha>rp$.

We also extend (1.4) to complete moment convergence, that is, we provide moment conditions under which

1.5 $$ \sum^{\infty}_{n=1}n^{r-2-q/p} E \Biggl( \max_{1\leq m\leq n} \Biggl\vert \sum^{m}_{k=1}a_{nk}X_{k} \Biggr\vert -\varepsilon n^{1/p} \Biggr)_{+}^{q}< \infty,\quad \forall\varepsilon>0, $$

where $q>0$.

Note that if (1.5) holds for some $q>0$, then (1.4) also holds. The proof is well known.

Throughout this paper, *C* always stands for a positive constant that may differ from one place to another. For events *A* and *B*, we denote $I(A, B)=I(A\cap B)$, where $I(A)$ is the indicator function of an event *A*.

## Preliminary lemmas

To prove the main results, we need the following lemmas. The first one belongs to Utev and Peligrad [6].

### Lemma 2.1

*Let* $q\geq2$, *and let* $\{X_{n}, n\ge1\}$ *be a sequence of* $\rho^{*}$-*mixing random variables with* $EX_{n}=0$ *and* $E|X_{n}|^{q}<\infty$ *for every* $n\ge1$. *Then for all* $n\ge1$,

$$E\max_{1\le m\le n} \Biggl\vert \sum_{k=1}^{m}X_{k} \Biggr\vert ^{q} \le C_{q} \Biggl\{ \sum _{k=1}^{n} E|X_{k}|^{q}+ \Biggl( \sum_{k=1}^{n}E|X_{k}|^{2} \Biggr)^{q/2} \Biggr\} , $$

*where* $C_{q}>0$ *depends only on* *q* *and the* $\rho^{*}$-*mixing coefficients*.

### Remark 2.1

By the Hölder inequality, (1.1) implies that

$$\sup_{n\geq1}n^{-1}\sum^{n}_{k=1}|a_{nk}|^{s}< \infty $$

for any $0< s\leq\alpha$, and

$$\sup_{n\geq1}n^{-q/\alpha}\sum^{n}_{k=1}|a_{nk}|^{q}< \infty $$

for any $q>\alpha$. These properties will be used in the proofs of the following lemmas and main results.

### Lemma 2.2

*Let* $r\geq1$, $0< p<2$, $\alpha>0$, $\beta>0$ *with* $1/\alpha+1/\beta=1/p$, *and let* *X* *be a random variable*. *Let* $\{a_{nk},1\leq k\leq n, n\geq1\}$ *be an array of constants satisfying* (1.1). *Then*

2.1 $$ \sum^{\infty}_{n=1}n^{r-2}\sum ^{n}_{k=1}P\bigl\{ \vert a_{nk}X \vert >n^{1/p}\bigr\} \leq \textstyle\begin{cases} C E \vert X \vert ^{(r-1)\beta} &\textit{if }\alpha< rp, \\ C E \vert X \vert ^{(r-1)\beta}\log(1+ \vert X \vert ) &\textit{if }\alpha=rp, \\ C E \vert X \vert ^{rp} &\textit{if }\alpha>rp. \end{cases} $$

### Proof

Case 1: $\alpha\leq rp$. We observe by the Markov inequality that, for any $s>0$,

2.2 $$\begin{aligned} &P\bigl\{ \vert a_{nk}X \vert >n^{1/p}\bigr\} \\ &\quad =P\bigl\{ \vert a_{nk}X \vert >n^{1/p}, \vert X \vert >n^{1/\beta}\bigr\} +P\bigl\{ \vert a_{nk}X \vert >n^{1/p}, \vert X \vert \leq n^{1/\beta}\bigr\} \\ &\quad \leq n^{-\alpha/p} \vert a_{nk} \vert ^{\alpha}E \vert X \vert ^{\alpha}I\bigl( \vert X \vert >n^{1/\beta } \bigr)+n^{-s/p} \vert a_{nk} \vert ^{s}E \vert X \vert ^{s}I\bigl( \vert X \vert \leq n^{1/\beta}\bigr). \end{aligned}$$

It is easy to show that

2.3 $$\begin{aligned} &\sum^{\infty}_{n=1}n^{r-2}\cdot n^{-\alpha/p} \Biggl(\sum^{n}_{k=1} \vert a_{nk} \vert ^{\alpha}\Biggr)E \vert X \vert ^{\alpha}I\bigl( \vert X \vert >n^{1/\beta}\bigr) \\ &\quad \leq C\sum^{\infty}_{n=1}n^{r-1-\alpha/p}E \vert X \vert ^{\alpha}I\bigl( \vert X \vert >n^{1/\beta } \bigr) \\ &\quad \leq \textstyle\begin{cases} CE \vert X \vert ^{(r-1)\beta} &\text{if }\alpha< rp, \\ CE \vert X \vert ^{(r-1)\beta}\log(1+ \vert X \vert ) &\text{if }\alpha=rp. \end{cases}\displaystyle \end{aligned}$$

Taking $s>\max\{\alpha, (r-1)\beta\}$, we have that

2.4 $$\begin{aligned} &\sum^{\infty}_{n=1}n^{r-2}\cdot n^{-s/p} \Biggl(\sum^{n}_{k=1} \vert a_{nk} \vert ^{s} \Biggr)E \vert X \vert ^{s}I\bigl( \vert X \vert \leq n^{1/\beta}\bigr) \\ &\quad \leq C\sum^{\infty}_{n=1}n^{r-2-s/p+s/\alpha}E \vert X \vert ^{s}I\bigl( \vert X \vert \leq n^{1/\beta } \bigr) \\ &\quad =C\sum^{\infty}_{n=1}n^{r-2-s/\beta}E \vert X \vert ^{s}I\bigl( \vert X \vert \leq n^{1/\beta} \bigr) \\ &\quad \leq CE \vert X \vert ^{(r-1)\beta}, \end{aligned}$$

since $s>(r-1)\beta$. Then (2.1) holds by (2.2)–(2.4).

Case 2: $\alpha>rp$. The proof is similar to that of Case 1. However, we use a different truncation for *X*. We observe by the Markov inequality that, for any $t>0$,

2.5 $$\begin{aligned} &P\bigl\{ \vert a_{nk}X \vert >n^{1/p}\bigr\} \\ &\quad =P\bigl\{ \vert a_{nk}X \vert >n^{1/p}, \vert X \vert >n^{1/p}\bigr\} +P\bigl\{ \vert a_{nk}X \vert >n^{1/p}, \vert X \vert \leq n^{1/p}\bigr\} \\ &\quad \leq n^{-t/p} \vert a_{nk} \vert ^{t} E \vert X \vert ^{t} I\bigl( \vert X \vert >n^{1/p} \bigr)+n^{-\alpha /p} \vert a_{nk} \vert ^{\alpha}E \vert X \vert ^{\alpha}I\bigl( \vert X \vert \leq n^{1/p}\bigr). \end{aligned}$$

Taking $0< t< rp$, we have that

2.6 $$\begin{aligned} &\sum^{\infty}_{n=1}n^{r-2}\cdot n^{-t/p} \Biggl(\sum^{n}_{k=1} \vert a_{nk} \vert ^{t} \Biggr)E \vert X \vert ^{t}I\bigl( \vert X \vert >n^{1/p}\bigr) \\ &\quad \leq C\sum^{\infty}_{n=1}n^{r-1-t/p}E \vert X \vert ^{t}I\bigl( \vert X \vert >n^{1/p}\bigr) \\ &\quad \leq CE \vert X \vert ^{rp}. \end{aligned}$$

It is easy to show that

2.7 $$\begin{aligned} &\sum^{\infty}_{n=1}n^{r-2}\cdot n^{-\alpha/p} \Biggl(\sum^{n}_{k=1} \vert a_{nk} \vert ^{\alpha}\Biggr)E \vert X \vert ^{\alpha}I\bigl( \vert X \vert \leq n^{1/p}\bigr) \\ &\quad \leq C\sum^{\infty}_{n=1}n^{r-1-\alpha/p}E \vert X \vert ^{\alpha}I\bigl( \vert X \vert \leq n^{1/p} \bigr) \\ &\quad \leq CE \vert X \vert ^{rp}, \end{aligned}$$

since $\alpha>rp$. Then (2.1) holds by (2.5)–(2.7). □

### Lemma 2.3

*Let* $r\geq1$, $0< p<2$, $\alpha>0$, $\beta>0$ *with* $1/\alpha+1/\beta=1/p$, *and let* *X* *be a random variable*. *Let* $\{a_{nk},1\leq k\leq n, n\geq1\}$ *be an array of constants satisfying* (1.1). *Then*, *for any* $s>\max\{\alpha, (r-1)\beta\}$,

2.8 $$\begin{aligned}& \sum^{\infty}_{n=1}n^{r-2-s/p}\sum ^{n}_{k=1}E \vert a_{nk}X \vert ^{s}I\bigl( \vert a_{nk}X \vert \leq n^{1/p} \bigr) \\& \quad \leq \textstyle\begin{cases} C E|X|^{(r-1)\beta} &\textit{if }\alpha< rp, \\ C E|X|^{(r-1)\beta}\log(1+|X|) &\textit{if }\alpha=rp, \\ C E|X|^{rp} &\textit{if }\alpha>rp. \end{cases}\displaystyle \end{aligned}$$

### Proof

Case 1: $\alpha\leq rp$. By (2.3) and (2.4) we get that

$$\begin{aligned} &\sum^{\infty}_{n=1}n^{r-2-s/p}\sum ^{n}_{k=1}E \vert a_{nk}X \vert ^{s}I\bigl( \vert a_{nk}X \vert \leq n^{1/p} \bigr) \\ &\quad =\sum^{\infty}_{n=1}n^{r-2-s/p}\sum ^{n}_{k=1}E \vert a_{nk}X \vert ^{s}I\bigl( \vert a_{nk}X \vert \leq n^{1/p}, \vert X \vert >n^{1/\beta}\bigr) \\ &\qquad {} +\sum^{\infty}_{n=1}n^{r-2-s/p} \sum^{n}_{k=1}E \vert a_{nk}X \vert ^{s}I\bigl( \vert a_{nk}X \vert \leq n^{1/p}, \vert X \vert \leq n^{1/\beta}\bigr) \\ &\quad \leq\sum^{\infty}_{n=1}n^{r-2-s/p}n^{(s-\alpha)/p} \sum^{n}_{k=1}E \vert a_{nk}X \vert ^{\alpha}I\bigl( \vert X \vert >n^{1/\beta}\bigr) \\ &\qquad {} +\sum^{\infty}_{n=1}n^{r-2-s/p} \sum^{n}_{k=1}E \vert a_{nk}X \vert ^{s}I\bigl( \vert X \vert \leq n^{1/\beta}\bigr) \\ &\quad \leq \textstyle\begin{cases} C E \vert X \vert ^{(r-1)\beta} &\text{if }\alpha< rp, \\ C E \vert X \vert ^{(r-1)\beta}\log(1+ \vert X \vert ) &\text{if }\alpha=rp. \end{cases}\displaystyle \end{aligned}$$

Case 2: $\alpha>rp$. Taking $0< t< rp$, we have by (2.6) and (2.7) that

$$\begin{aligned} &\sum^{\infty}_{n=1}n^{r-2-s/p}\sum ^{n}_{k=1}E \vert a_{nk}X \vert ^{s}I\bigl( \vert a_{nk}X \vert \leq n^{1/p} \bigr) \\ &\quad = \sum^{\infty}_{n=1}n^{r-2-s/p} \sum^{n}_{k=1}E \vert a_{nk}X \vert ^{s}I\bigl( \vert a_{nk}X \vert \leq n^{1/p}, \vert X \vert >n^{1/p}\bigr) \\ &\qquad {} +\sum^{\infty}_{n=1}n^{r-2-s/p} \sum^{n}_{k=1}E \vert a_{nk}X \vert ^{s}I\bigl( \vert a_{nk}X \vert \leq n^{1/p}, \vert X \vert \leq n^{1/p}\bigr) \\ &\quad \leq\sum^{\infty}_{n=1}n^{r-2-s/p}n^{(s-t)/p} \sum^{n}_{k=1}E \vert a_{nk}X \vert ^{t}I\bigl( \vert X \vert >n^{1/p}\bigr) \\ &\qquad {} +\sum^{\infty}_{n=1}n^{r-2-s/p}n^{(s-\alpha)/p} \sum^{n}_{k=1}E \vert a_{nk}X \vert ^{\alpha} I\bigl( \vert X \vert \leq n^{1/p}\bigr) \\ &\quad \leq CE \vert X \vert ^{rp}. \end{aligned}$$

Therefore (2.8) holds. □

The following lemma is a counterpart of Lemma 2.3. The truncation for $|a_{nk}X|$ is reversed.

### Lemma 2.4

*Let* $q>0$, $r\geq1$, $0< p<2$, $\alpha>0$, $\beta>0$ *with* $1/\alpha+1/\beta=1/p$, *and let* *X* *be a random variable*. *Let* $\{a_{nk},1\leq k\leq n, n\geq1\}$ *be an array of constants satisfying* (1.1). *Then the following statements hold*.

1. *If* $\alpha< rp$, *then*
  2.9 $$\begin{aligned}& \sum^{\infty}_{n=1}n^{r-2-q/p}\sum ^{n}_{k=1}E \vert a_{nk}X \vert ^{q}I\bigl( \vert a_{nk}X \vert >n^{1/p}\bigr) \\& \quad \leq \textstyle\begin{cases} C E|X|^{(r-1)\beta} &\textit{if }q< (r-1)\beta, \\ C E|X|^{(r-1)\beta}\log(1+|X|) &\textit{if }q=(r-1)\beta, \\ C E|X|^{q} &\textit{if }q>(r-1)\beta. \end{cases}\displaystyle \end{aligned}$$
2. *If* $\alpha=rp$, *then*
  2.10 $$\begin{aligned}& \sum^{\infty}_{n=1}n^{r-2-q/p}\sum ^{n}_{k=1}E \vert a_{nk}X \vert ^{q}I\bigl( \vert a_{nk}X \vert >n^{1/p}\bigr) \\& \quad \leq \textstyle\begin{cases} C E|X|^{(r-1)\beta}\log(1+|X|) &\textit{if }q\leq\alpha=rp, \\ C E|X|^{q} &\textit{if }q>\alpha=rp. \end{cases}\displaystyle \end{aligned}$$
3. *If* $\alpha>rp$, *then*
  2.11 $$\begin{aligned} \begin{aligned}[b] &\sum^{\infty}_{n=1}n^{r-2-q/p}\sum ^{n}_{k=1}E \vert a_{nk}X \vert ^{q}I\bigl( \vert a_{nk}X \vert >n^{1/p}\bigr) \\ &\quad \leq \textstyle\begin{cases} C E|X|^{rp} &\textit{if }q< rp, \\ C E|X|^{rp}\log(1+|X|) &\textit{if }q=rp, \\ C E|X|^{q} &\textit{if }q>rp. \end{cases}\displaystyle \end{aligned} \end{aligned}$$

### Proof

Without loss of generality, we may assume that $n^{-1}\sum_{k=1}^{n} |a_{nk}|^{\alpha}\leq1$ for all $n\ge1$. From this we have that $|a_{nk}|\leq n^{1/\alpha}$ for all $1\leq k\leq n$ and $n\geq1$.

(1) In this case, we have that $\alpha< rp<(r-1)\beta$. If $0< q<\alpha$, then

2.12 $$\begin{aligned} &\sum^{\infty}_{n=1}n^{r-2-q/p}\sum _{k=1}^{n} E \vert a_{nk}X \vert ^{q}I\bigl( \vert a_{nk}X \vert >n^{1/p} \bigr) \\ &\quad \leq\sum^{\infty}_{n=1}n^{r-2-q/p}n^{-(\alpha-q)/p} \sum_{k=1}^{n} E \vert a_{nk}X \vert ^{\alpha}I\bigl( \vert a_{nk}X \vert >n^{1/p}\bigr) \\ &\quad \leq\sum^{\infty}_{n=1}n^{r-2-q/p}n^{-(\alpha-q)/p} \sum_{k=1}^{n} E \vert a_{nk}X \vert ^{\alpha}I\bigl( \bigl\vert n^{1/\alpha}X \bigr\vert >n^{1/p}\bigr) \\ &\quad \leq C\sum^{\infty}_{n=1}n^{r-1-\alpha/p} E \vert X \vert ^{\alpha}I\bigl( \vert X \vert >n^{1/\beta } \bigr) \\ &\quad =C\sum_{i=1}^{\infty}E \vert X \vert ^{\alpha}I\bigl(i^{1/\beta}< \vert X \vert \le(i+1)^{1/\beta }\bigr)\sum_{n=1}^{i} n^{r-1-\alpha/p} \\ &\quad \leq C E \vert X \vert ^{(r-1)\beta}. \end{aligned}$$

If $q\geq\alpha$, then

2.13 $$\begin{aligned} &\sum^{\infty}_{n=1}n^{r-2-q/p}\sum _{k=1}^{n} E \vert a_{nk}X \vert ^{q}I\bigl( \vert a_{nk}X \vert >n^{1/p} \bigr) \\ &\quad \leq\sum^{\infty}_{n=1}n^{r-2-q/p} \sum_{k=1}^{n} E \vert a_{nk}X \vert ^{q}I\bigl( \bigl\vert n^{1/\alpha}X \bigr\vert >n^{1/p}\bigr) \\ &\quad =\sum^{\infty}_{n=1}n^{r-2-q/p} \sum_{k=1}^{n} E \vert a_{nk}X \vert ^{q}I\bigl( \vert X \vert >n^{1/\beta }\bigr) \\ &\quad \leq C\sum^{\infty}_{n=1}n^{r-2-q/p+q/\alpha} E \vert X \vert ^{q}I\bigl( \vert X \vert >n^{1/\beta} \bigr) \\ &\quad =C\sum_{i=1}^{\infty}E \vert X \vert ^{q}I\bigl(i^{1/\beta}< \vert X \vert \le(i+1)^{1/\beta}\bigr)\sum_{n=1}^{i} n^{r-2-q/\beta} \\ &\quad \leq \textstyle\begin{cases} C E \vert X \vert ^{(r-1)\beta} &\text{if }\alpha\le q< (r-1)\beta, \\ C E \vert X \vert ^{(r-1)\beta}\log(1+ \vert X \vert ) &\text{if }q=(r-1)\beta, \\ C E \vert X \vert ^{q} &\text{if }q>(r-1)\beta. \end{cases}\displaystyle \end{aligned}$$

Combining (2.12) and (2.13) gives (2.9).

(2) In this case, we have that $\alpha=rp=(r-1)\beta$. If $q\le\alpha=rp=(r-1)\beta$, then

2.14 $$\begin{aligned} &\sum^{\infty}_{n=1}n^{r-2-q/p}\sum _{k=1}^{n} E \vert a_{nk}X \vert ^{q}I\bigl( \vert a_{nk}X \vert >n^{1/p} \bigr) \\ &\quad \leq\sum^{\infty}_{n=1}n^{r-2-q/p+(q-\alpha)/p} \sum^{n}_{k=1}E \vert a_{nk}X \vert ^{\alpha}I\bigl( \bigl\vert n^{1/\alpha}X \bigr\vert >n^{1/p}\bigr) \\ &\quad \leq C \sum^{\infty}_{n=1}n^{r-1-\alpha/p}E \vert X \vert ^{\alpha}I\bigl( \vert X \vert >n^{\beta}\bigr) \\ &\quad =C\sum^{\infty}_{n=1}n^{-1}E \vert X \vert ^{\alpha}I\bigl( \vert X \vert >n^{\beta}\bigr) \\ &\quad =C\sum_{i=1}^{\infty}E \vert X \vert ^{\alpha}I\bigl(i^{\beta}< \vert X \vert \le(i+1)^{\beta}\bigr)\sum_{n=1}^{i} n^{-1} \\ &\quad \leq CE \vert X \vert ^{(r-1)\beta}\log\bigl(1+ \vert X \vert \bigr). \end{aligned}$$

If $q>\alpha=rp=(r-1)\beta$, then

2.15 $$\begin{aligned} &\sum^{\infty}_{n=1}n^{r-2-q/p}\sum _{k=1}^{n} E \vert a_{nk}X \vert ^{q}I\bigl( \vert a_{nk}X \vert >n^{1/p} \bigr) \\ &\quad \le C\sum^{\infty}_{n=1}n^{r-2-q/p}n^{q/\alpha} E \vert X \vert ^{q} \le CE \vert X \vert ^{q}. \end{aligned}$$

Combining (2.14) and (2.15) gives (2.10).

(3) In this case, we have that $(r-1)\beta< rp<\alpha$. If $q\le rp$, then

2.16 $$\begin{aligned} &\sum^{\infty}_{n=1}n^{r-2-q/p}\sum _{k=1}^{n} E \vert a_{nk}X \vert ^{q}I\bigl( \vert a_{nk}X \vert >n^{1/p} \bigr) \\ &\quad =\sum^{\infty}_{n=1}n^{r-2-q/p}\sum _{k=1}^{n} E \vert a_{nk}X \vert ^{q}I\bigl( \vert a_{nk}X \vert >n^{1/p}, \vert X \vert >n^{1/p}\bigr) \\ &\qquad {} +\sum^{\infty}_{n=1}n^{r-2-q/p} \sum_{k=1}^{n} E \vert a_{nk}X \vert ^{q}I\bigl( \vert a_{nk}X \vert >n^{1/p}, \vert X \vert \leq n^{1/p}\bigr) \\ &\quad \leq\sum^{\infty}_{n=1}n^{r-2-q/p} \sum_{k=1}^{n} E \vert a_{nk}X \vert ^{q} I\bigl( \vert X \vert >n^{1/p}\bigr) \\ &\qquad {} +\sum^{\infty}_{n=1}n^{r-2-q/p}n^{-(\alpha-q)/p} \sum_{k=1}^{n} E \vert a_{nk}X \vert ^{\alpha}I\bigl( \vert X \vert \leq n^{1/p}\bigr) \\ &\quad \leq C\sum^{\infty}_{n=1}n^{r-1-q/p} E \vert X \vert ^{q} I\bigl( \vert X \vert >n^{1/p} \bigr) \\ &\qquad {} +C\sum^{\infty}_{n=1}n^{r-1-\alpha/p}E \vert X \vert ^{\alpha}I\bigl( \vert X \vert \leq n^{1/p} \bigr) \\ &\quad = C\sum_{i=1}^{\infty}E \vert X \vert ^{q} I\bigl(i^{1/p}< \vert X \vert \le(i+1)^{1/p}\bigr)\sum_{n=1}^{i} n^{r-1-q/p} \\ &\qquad {} +C\sum_{i=1}^{\infty}E \vert X \vert ^{\alpha}I\bigl((i-1)^{1/p}< \vert X \vert \le i^{1/p}\bigr)\sum_{n=i}^{\infty}n^{r-1-\alpha/p} \\ &\quad \leq \textstyle\begin{cases} C E \vert X \vert ^{rp} &\text{if }q< rp, \\ C E \vert X \vert ^{rp}\log(1+ \vert X \vert ) &\text{if }q=rp. \end{cases}\displaystyle \end{aligned}$$

If $rp< q<\alpha$, then

2.17 $$\begin{aligned} &\sum^{\infty}_{n=1}n^{r-2-q/p}\sum _{k=1}^{n} E \vert a_{nk}X \vert ^{q}I\bigl( \vert a_{nk}X \vert >n^{1/p} \bigr) \\ &\quad \le C\sum^{\infty}_{n=1}n^{r-1-q/p} E \vert X \vert ^{q} \le CE \vert X \vert ^{q}. \end{aligned}$$

If $q\ge\alpha$, then

2.18 $$\begin{aligned} &\sum^{\infty}_{n=1}n^{r-2-q/p}\sum _{k=1}^{n} E \vert a_{nk}X \vert ^{q}I\bigl( \vert a_{nk}X \vert >n^{1/p} \bigr) \\ &\quad \le C\sum^{\infty}_{n=1}n^{r-2-q/p}n^{q/\alpha} E \vert X \vert ^{q} \le CE \vert X \vert ^{q}, \end{aligned}$$

since $q\ge\alpha>(r-1)\beta$.

Combining (2.16)–(2.18) gives (2.11). □

### Lemma 2.5

*Let* $1\leq p<2$, $\alpha>0$, $\beta>0$ *with* $1/\alpha +1/\beta=1/p$, *and let* *X* *be a random variable*. *Let* $\{a_{nk},1\leq k\leq n, n\geq1\}$ *be an array of constants satisfying* (1.1). *If* $E|X|^{p}<\infty$, *then*

2.19 $$ n^{-1/p}\sum^{n}_{k=1}E \vert a_{nk}X \vert I\bigl( \vert a_{nk}X \vert > n^{1/p}\bigr)\rightarrow0 $$

*as* $n\rightarrow\infty$, *and hence*, *in addition*, *if* $EX=0$, *then*

2.20 $$ n^{-1/p}\max_{1\leq m\leq n} \Biggl\vert \sum ^{m}_{k=1}a_{nk}EXI\bigl( \vert a_{nk}X \vert \leq n^{1/p}\bigr) \Biggr\vert \rightarrow0 $$

*as* $n\rightarrow\infty$.

### Proof

Denote $A^{\alpha}=\sup_{n\geq1}n^{-1}\sum^{n}_{k=1}|a_{nk}|^{\alpha}$. Then $|a_{nk}|\leq An^{1/\alpha}$ for all $1\leq k\leq n$ and $n\geq1$. It follows that

2.21 $$\begin{aligned} &n^{-1/p}\sum^{n}_{k=1}E \vert a_{nk}X \vert I\bigl( \vert a_{nk}X \vert >n^{1/p}\bigr) \\ &\quad \leq n^{-1}\sum^{n}_{k=1}E \vert a_{nk}X \vert ^{p}I\bigl( \vert a_{nk}X \vert >n^{1/p}\bigr) \\ &\quad \leq n^{-1} \Biggl(\sum^{n}_{k=1} \vert a_{nk} \vert ^{p} \Biggr)E \vert X \vert ^{p}I\bigl( \vert AX \vert >n^{1/\beta }\bigr) \\ &\quad \leq CE \vert X \vert ^{p}I\bigl( \vert AX \vert >n^{1/\beta}\bigr)\rightarrow0 \end{aligned}$$

as $n\rightarrow\infty$. Hence (2.19) holds.

If, in addition, $EX=0$, then we get by (2.21) that

$$\begin{aligned} &n^{-1/p}\max_{1\leq m\leq n} \Biggl\vert \sum ^{m}_{k=1}a_{nk}EXI\bigl( \vert a_{nk}X \vert \leq n^{1/p}\bigr) \Biggr\vert \\ &\quad =n^{-1/p}\max_{1\leq m\leq n} \Biggl\vert \sum ^{m}_{k=1}a_{nk}EXI\bigl( \vert a_{nk}X \vert > n^{1/p}\bigr) \Biggr\vert \\ &\quad \le n^{-1/p}\sum^{n}_{k=1}E \vert a_{nk}X \vert I\bigl( \vert a_{nk}X \vert >n^{1/p}\bigr)\rightarrow0 \end{aligned}$$

as $n\rightarrow\infty$. Hence (2.20) holds. □

The following lemma shows that if $0< p<1$, then (2.20) holds without the condition $EX=0$.

### Lemma 2.6

*Let* $0< p<1$, $\alpha>0$, $\beta>0$ *with* $1/\alpha +1/\beta=1/p$, *and let* *X* *be a random variable*. *Let* $\{a_{nk},1\leq k\leq n, n\geq1\}$ *be an array of constants satisfying* (1.1). *If* $E|X|^{p}<\infty$, *then*

$$n^{-1/p}\sum^{n}_{k=1}E \vert a_{nk}X \vert I\bigl( \vert a_{nk}X \vert \le n^{1/p}\bigr)\rightarrow0 $$

*as* $n\rightarrow\infty$, *and hence* (2.20) *holds*.

### Proof

Note that

$$\begin{aligned} &n^{-1/p}\sum^{n}_{k=1}E \vert a_{nk}X \vert I\bigl( \vert a_{nk}X \vert \le n^{1/p}\bigr) \\ &\quad =n^{-1/p}\sum^{n}_{k=1}E \vert a_{nk}X \vert I\bigl( \vert a_{nk}X \vert \le n^{1/p}, \vert X \vert > n^{1/\beta }\bigr) \\ &\qquad {} +n^{-1/p}\sum^{n}_{k=1}E \vert a_{nk}X \vert I\bigl( \vert a_{nk}X \vert \le n^{1/p}, \vert X \vert \le n^{1/\beta}\bigr) \\ &\quad \le n^{-1/p}n^{(1-p)/p} \sum^{n}_{k=1}E \vert a_{nk}X \vert ^{p}I\bigl( \vert X \vert > n^{1/\beta }\bigr)+n^{-1/p}\sum^{n}_{k=1}E \vert a_{nk}X \vert I\bigl( \vert X \vert \le n^{1/\beta} \bigr) \\ &\quad \le C E \vert X \vert ^{p}I\bigl( \vert X \vert > n^{1/\beta}\bigr)+Cn^{-1/p+1/(\alpha\wedge1)} E \vert X \vert I\bigl( \vert X \vert \le n^{1/\beta}\bigr) \\ &\quad \le C E \vert X \vert ^{p}I\bigl( \vert X \vert > n^{1/\beta}\bigr)+Cn^{-1/p+1/(\alpha\wedge1)+ (1-p)/\beta}E \vert X \vert ^{p} \rightarrow0 \end{aligned}$$

as $n\rightarrow\infty$, since $-1/p+1/(\alpha\wedge1)+ (1-p)/\beta =-p/\beta$ if $\alpha\le1$ and $-1/p+1/(\alpha\wedge1)+ (1-p)/\beta =-(1-p)/\alpha$ if $\alpha>1$. □

## Main results

We first present complete convergence for weighted sums of $\rho ^{*}$-mixing random variables.

### Theorem 3.1

*Let* $r\geq1$, $1\leq p<2$, $\alpha>0$, $\beta>0$ *with* $1/\alpha+1/\beta=1/p$. *Let* $\{a_{nk}, 1\leq k\leq n, n\geq1\}$ *be an array of constants satisfying* (1.1), *and let* $\{X,X_{n},n\geq1\}$ *be a sequence of identically distributed* $\rho^{*}$-*mixing random variables*. *If*

3.1 $$ EX=0, \qquad \textstyle\begin{cases} E|X|^{(r-1)\beta}< \infty&\textit{if }\alpha< rp, \\ E|X|^{(r-1)\beta}\log(1+|X|)< \infty&\textit{if }\alpha=rp, \\ E|X|^{rp}< \infty&\textit{if }\alpha>rp, \end{cases} $$

*then* (1.4) *holds*.

*Conversely*, *if* (1.4) *holds for any array* $\{a_{nk}, 1\le k\le n,n\ge 1\}$ *satisfying* (1.1) *for some* $\alpha>p$, *then* $EX=0$, $E|X|^{rp}<\infty$ *and* $E|X|^{(r-1)\beta}<\infty$.

### Remark 3.1

When $0< p<1$, (3.1) without the condition $EX=0$ implies (1.4). The proof is the same as that of Theorem 3.1 except that Lemma 2.5 is replaced by Lemma 2.6.

### Remark 3.2

The case $\alpha>rp$ $(r>1)$ of Theorem 3.1 corresponds to Theorem 2.2 of Sung [1], and the proof is much simpler than that of Sung [1]. Hence Theorem 3.1 generalizes the result of Sung [1].

### Remark 3.3

Suppose that $r\ge1$, $1\leq p<2$, $\alpha>0$, $\beta >0$ with $1/\alpha+1/\beta=1/p$. Then the case $\alpha< rp$ is equivalent to the case $rp<(r-1)\beta$, and in this case, $\alpha< rp<(r-1)\beta$. The case $\alpha=rp$ is equivalent to the case $rp=(r-1)\beta$, and in this case, $\alpha=rp=(r-1)\beta$. The case $\alpha>rp$ is equivalent to the case $rp>(r-1)\beta$, and in this case, $\alpha>rp>(r-1)\beta$.

### Remark 3.4

In two cases $\alpha< rp$ and $\alpha>rp$, the moment conditions are necessary and sufficient conditions, but in the case $\alpha=rp$, the moment condition $E|X|^{(r-1)\beta}\log (1+|X|)=E|X|^{rp}\log(1+|X|)<\infty$ is only sufficient for (1.4). It may be difficult to prove (1.4) under the necessary moment condition $E|X|^{rp}<\infty$. An and Yuan [17] proved (1.4) under the moment condition $E|X|^{rp}<\infty$ and the condition

$$\sup_{n\geq1}n^{-\delta}\sum^{n}_{k=1}|a_{nk}|^{rp}< \infty $$

for some $\delta\in(0,1)$. However, their result is not an extension of the classical one and is a particular case of Sung [1]. In fact, if we set $\alpha =rp/\delta$, then $\alpha>rp$, and (1.1) holds.

### Proof of Theorem 3.1

Sufficiency. For any $1\leq k\leq n$ and $n\geq1$, set

$$X_{nk}=a_{nk}X_{k} I\bigl( \vert a_{nk}X_{k} \vert \leq n^{1/p}\bigr). $$

Note that

$$\Biggl\{ \max_{1\leq m\leq n} \Biggl\vert \sum ^{m}_{k=1}a_{nk}X_{k} \Biggr\vert >\varepsilon n^{1/p} \Biggr\} \subset\cup^{n}_{k=1} \bigl\{ \vert a_{nk}X_{k} \vert >n^{1/p}\bigr\} \cup \Biggl\{ \max_{1\leq m\leq n} \Biggl\vert \sum ^{m}_{k=1}X_{nk} \Biggr\vert >\varepsilon n^{1/p} \Biggr\} . $$

Then by Lemmas 2.2 and 2.5, to prove (1.4), it suffices to prove that

3.2 $$ \sum^{\infty}_{n=1}n^{r-2}P \Biggl\{ \max_{1\leq m\leq n} \Biggl\vert \sum^{m}_{k=1}(X_{nk}-EX_{nk}) \Biggr\vert >\varepsilon n^{1/p} \Biggr\} < \infty,\quad \forall \varepsilon>0. $$

When $r>1$, set $s\in(p, \min\{2, \alpha\})$ if $\alpha\leq rp$ and $s\in(p, \min\{2, rp\})$ if $\alpha>rp$. Note that, when $r=1$, we cannot choose such *s*, since $\alpha>p=rp$. Then $p< s<\min\{2,\alpha\}$, and $E|X|^{s}<\infty$ by Remark 3.3. Taking $q>\max\{2,\alpha, (r-1)\beta, 2p(r-1)/(s-p)\}$, we have by the Markov inequality and Lemma 2.1 that

3.3 $$\begin{aligned} &P\Biggl\{ \max_{1\leq m\leq n} \Biggl\vert \sum ^{m}_{k=1}(X_{nk}-EX_{nk}) \Biggr\vert >\varepsilon n^{1/p}\Biggr\} \\ &\quad \leq Cn^{-q/p} \Biggl(\sum^{n}_{k=1}E(X_{nk}-EX_{nk})^{2} \Biggr)^{q/2}+Cn^{-q/p}\sum^{n}_{k=1}E \vert X_{nk}-EX_{nk} \vert ^{q}. \end{aligned}$$

Since $q>2p(r-1)/(s-p)$, we have that $r-2+q(1-s/p)/2<-1$. It follows that

3.4 $$\begin{aligned} &\sum^{\infty}_{n=1}n^{r-2}\cdot n^{-q/p} \Biggl(\sum^{n}_{k=1}E(X_{nk}-EX_{nk})^{2} \Biggr)^{q/2} \\ &\quad \leq\sum^{\infty}_{n=1}n^{r-2} \cdot n^{-q/p} \Biggl(\sum^{n}_{k=1}E \vert a_{nk}X_{k} \vert ^{2}I\bigl( \vert a_{nk}X_{k} \vert \leq n^{1/p}\bigr) \Biggr)^{q/2} \\ &\quad \leq\sum^{\infty}_{n=1}n^{r-2} \cdot n^{-q/p} \Biggl(n^{(2-s)/p}\sum^{n}_{k=1}E \vert a_{nk}X_{k} \vert ^{s}I\bigl( \vert a_{nk}X_{k} \vert \leq n^{1/p}\bigr) \Biggr)^{q/2} \\ &\quad \leq\sum^{\infty}_{n=1}n^{r-2} \cdot n^{-q/p} \Biggl(n^{(2-s)/p}\sum^{n}_{k=1} \vert a_{nk} \vert ^{s}E \vert X \vert ^{s} \Biggr)^{q/2} \\ &\quad \leq C\sum^{\infty}_{n=1}n^{r-2+q(1-s/p)/2}< \infty. \end{aligned}$$

By Lemma 2.3 we have

3.5 $$\begin{aligned} &\sum^{\infty}_{n=1}n^{r-2}\cdot n^{-q/p}\sum^{n}_{k=1}E \vert X_{nk}-EX_{nk} \vert ^{q} \\ &\quad \leq C\sum^{\infty}_{n=1}n^{r-2} \cdot n^{-q/p}\sum^{n}_{k=1}E \vert a_{nk}X_{k} \vert ^{q}I\bigl( \vert a_{nk}X_{k} \vert \leq n^{1/p}\bigr) \\ &\quad < \infty. \end{aligned}$$

Hence (3.2) holds by (3.3)–(3.5).

When $r=1$, we always have that $\alpha>p=rp$. If (1.1) holds for some $\alpha>0$, then (1.1) also holds for any $\alpha'$ ($0<\alpha'\leq\alpha$) by Remark 2.1. Thus we may assume that $p<\alpha<2$. Taking $q=2$, we have by the Markov inequality and Lemmas 2.1 and 2.3 that

$$\begin{aligned} &\sum^{\infty}_{n=1}n^{r-2}P \Biggl\{ \max_{1\leq m\leq n} \Biggl\vert \sum^{m}_{k=1}(X_{nk}-EX_{nk}) \Biggr\vert >\varepsilon n^{1/p} \Biggr\} \\ &\quad \leq C\sum^{\infty}_{n=1}n^{r-2} \cdot n^{-2/p}\sum^{n}_{k=1}E \vert a_{nk}X_{k} \vert ^{2}I\bigl( \vert a_{nk}X_{k} \vert \leq n^{1/p}\bigr) \\ &\quad < \infty. \end{aligned}$$

Necessity. Set $a_{nk}=1$ for all $1\leq k\leq n$ and $n\geq1$. Then (1.4) can be rewritten as

$$\sum^{\infty}_{n=1}n^{r-2}P\Biggl\{ \max_{1\leq m\leq n} \Biggl\vert \sum^{m}_{k=1}X_{k} \Biggr\vert >\varepsilon n^{1/p}\Biggr\} < \infty, \quad \forall \varepsilon>0, $$

which implies that $EX=0$ and $E|X|^{rp}<\infty$ (see Theorem 2 in Peligrad and Gut [5]). Set $a_{nk}=0$ if $1\leq k\leq n-1$ and $a_{nn}=n^{1/\alpha}$. Then (1.4) can be rewritten as

$$\sum^{\infty}_{n=1}n^{r-2}P\bigl\{ n^{1/\alpha} \vert X_{n} \vert >\varepsilon n^{1/p} \bigr\} < \infty, \quad \forall \varepsilon>0, $$

which is equivalent to $E|X|^{(r-1)\beta}<\infty$. The proof is completed. □

Now we extend Theorem 3.1 to complete moment convergence.

### Theorem 3.2

*Let* $q>0$, $r\geq1$, $1\leq p<2$, $\alpha>0$, $\beta>0$ *with* $1/\alpha+1/\beta=1/p$. *Let* $\{a_{nk}, 1\leq k\leq n, n\geq1\}$ *be an array of constants satisfying* (1.1), *and let* $\{X,X_{n},n\geq1\}$ *be a sequence of identically distributed* $\rho^{*}$-*mixing random variables*. *Assume that one of the following conditions holds*.

1. *If* $\alpha< rp$, *then*
  3.6 $$ EX=0,\qquad \textstyle\begin{cases} E|X|^{(r-1)\beta}< \infty &\textit{if }q< (r-1)\beta, \\ E|X|^{(r-1)\beta}\log(1+|X|)< \infty &\textit{if }q=(r-1)\beta, \\ E|X|^{q}< \infty &\textit{if }q>(r-1)\beta. \end{cases} $$
2. *If* $\alpha=rp$, *then*
  3.7 $$ EX=0,\qquad \textstyle\begin{cases} E|X|^{(r-1)\beta}\log(1+|X|)< \infty &\textit{if } q\leq\alpha=rp, \\ E|X|^{q}< \infty &\textit{if }q>\alpha=rp. \end{cases} $$
3. *If* $\alpha>rp$, *then*
  3.8 $$ EX=0,\qquad \textstyle\begin{cases} E|X|^{rp}< \infty &\textit{if }q< rp, \\ E|X|^{rp}\log(1+|X|)< \infty &\textit{if }q=rp, \\ E|X|^{q}< \infty &\textit{if }q>rp. \end{cases} $$

*Then* (1.5) *holds*.

### Remark 3.5

As stated in the Introduction, if (1.5) holds for some $q>0$, then (1.4) also holds. If $\alpha< rp$, $EX=0$, and $E|X|^{(r-1)\beta}<\infty$, then (3.6) holds for some $0< q<(r-1)\beta$. If $\alpha=rp$, $EX=0$, and $E|X|^{(r-1)\beta}\log(1+|X|)<\infty$, then (3.7) holds for some $0< q\le\alpha$. If $\alpha>rp$, $EX=0$, and $E|X|^{rp}<\infty$, then (3.8) holds for some $0< q< rp$. Therefore the sufficiency of Theorem 3.1 holds by Theorem 3.2.

### Remark 3.6

The case $\alpha>rp$ of Theorem 3.2 corresponds to combining Theorems 3.1 and 3.2 in Wu et al. [2]. The condition on weights $\{a_{nk}\}$ in Wu et al. [2] is

$$\sup_{n\ge1} n^{-1}\sum_{k=1}^{n} |a_{nk}|^{t}< \infty\quad \text{for some }t>\max\{rp, q\}, $$

which is stronger than (1.1) with $\alpha>rp$. Hence Theorem 3.2 generalizes and improves the results of Wu et al. [2].

### Remark 3.7

In this paper, the $\rho^{*}$-mixing condition is only used in Lemma 2.1. Therefore our main results (Theorems 3.1 and 3.2) also hold for random variables satisfying Lemma 2.1.

### Proof of Theorem 3.2

We apply Theorems 2.1 and 2.2 in Sung [18] with $X_{nk}=a_{nk}X_{k}$, $b_{n}=n^{r-2}$, $a_{n}=n^{1/p}$. When the second moment of *X* does not exist, we apply Theorem 2.1 in Sung [18]. We can easily prove that Theorem 2.1 in Sung [18] still holds for $0< q<1$. When the second moment of *X* exists, we apply Theorem 2.2 in Sung [18].

(1) If $\alpha< rp$, then $\alpha< rp<(r-1)\beta$ by Remark 3.3. We first consider the case $q<(r-1)\beta$. In this case, the moment conditions are $EX=0$ and $E|X|^{(r-1)\beta }<\infty$. When $q<(r-1)\beta<2$, we prove (1.5) by using Theorem 2.1 in Sung [18]. To apply Theorem 2.1 in Sung [18], we take $s=2$. By Lemma 2.1,

$$E\max_{1\le m\le n} \Biggl\vert \sum_{k=1}^{m} \bigl(X_{nk}'(x)- EX_{nk}'(x) \bigr) \Biggr\vert ^{2} \le C \sum_{k=1}^{n} E \bigl\vert X_{nk}'(x) \bigr\vert ^{2}, \quad \forall n\ge1, \forall x>0, $$

where $X_{nk}'(x)=a_{nk}X_{k}I(|a_{nk}X_{k}|\le x^{1/q})+x^{1/q}I(a_{nk}X_{k}>x^{1/q})-x^{1/q}I(a_{nk}X_{k}<-x^{1/q})$. By Lemma 2.3,

3.9 $$ \sum_{n=1}^{\infty}n^{r-2-s/p}\sum _{k=1}^{n} E \vert a_{nk}X_{k} \vert ^{s}I\bigl( \vert a_{nk}X_{k} \vert \le n^{1/p}\bigr)\le CE \vert X \vert ^{(r-1)\beta}< \infty. $$

By Lemma 2.4,

3.10 $$ \sum_{n=1}^{\infty}n^{r-2-q/p}\sum _{k=1}^{n} E \vert a_{nk}X_{k} \vert ^{q}I\bigl( \vert a_{nk}X_{k} \vert > n^{1/p}\bigr)\le CE \vert X \vert ^{(r-1)\beta}< \infty. $$

By Lemma 2.5 (note that $E|X|^{p}<\infty$, since $p\le rp<(r-1)\beta$),

3.11 $$ n^{-1/p}\sum_{k=1}^{n} E \vert a_{nk}X_{k} \vert I\bigl( \vert a_{nk}X_{k} \vert >n^{1/p}\bigr)\to0. $$

Hence all conditions of Theorem 2.1 in Sung [18] are satisfied. Therefore (1.5) holds by Theorem 2.1 in Sung [18].

When $q<(r-1)\beta$ and $(r-1)\beta\ge2$, we prove (1.5) by using Theorem 2.2 in Sung [18]. To apply Theorem 2.2 in Sung [18], we take $s>0$ such that $s>\max \{2,q,\alpha, (r-1)\beta, (r-1)p(\alpha\wedge2)/((\alpha\wedge2)-p)\}$. By Lemma 2.1,

$$\begin{aligned} &E\max_{1\le m\le n} \Biggl\vert \sum_{k=1}^{m} \bigl(X_{nk}'(x)- EX_{nk}'(x) \bigr) \Biggr\vert ^{s} \\ &\quad \le C \Biggl\{ \sum_{k=1}^{n} E \bigl\vert X_{nk}'(x) \bigr\vert ^{s} + \Biggl( \sum_{k=1}^{n} E \bigl\vert X_{nk}'(x) \bigr\vert ^{2} \Biggr)^{s/2} \Biggr\} ,\quad \forall n\ge1, \forall x>0. \end{aligned}$$

Since $s>\max\{\alpha, (r-1)\beta\}$, (3.9) holds. Also, (3.10) and (3.11) hold. Since $E|X|^{2}<\infty$ and $s>(r-1)p(\alpha\wedge2)/((\alpha\wedge 2)-p)$, we have that

$$\sum_{n=1}^{\infty}n^{r-2} \Biggl( n^{-2/p}\sum_{k=1}^{n} E \vert a_{nk}X_{k} \vert ^{2} \Biggr)^{s/2}\le C \sum_{n=1}^{\infty}n^{r-2} \bigl( n^{-2/p} n^{2/(\alpha\wedge2)} \bigr)^{s/2}< \infty. $$

Hence all conditions of Theorem 2.2 in Sung [18] are satisfied. Therefore (1.5) holds by Theorem 2.2 in Sung [18].

For the cases $q=(r-1)\beta$ and $q>(r-1)\beta$, the proofs are similar to that of the previous case and are omitted.

The proofs of (2) and (3) are similar to that of (1) and are omitted. □
